# Computational Study of Electron Delocalization in Hexaarylbenzenes

**DOI:** 10.3390/molecules19033274

**Published:** 2014-03-17

**Authors:** Citlalli Rios, Roberto Salcedo

**Affiliations:** Instituto de Investigaciones en Materiales, Universidad Nacional Autónoma de México, Circuito Exterior s/n, Ciudad Universitaria, 04510 Coyoacán, México, D.F., Mexico

**Keywords:** toroidal delocalization, hexaarylbenzenes, isodesmic reactions

## Abstract

A number of hexaarylbenzene compounds were studied theoretically, in order to compare energy changes as a result of the toroidal delocalization effect that is characteristic of all these species. The energy was studied taking advantage of locally designed isodesmic reactions. Results indicate that the amount of aromaticity manifested by each substituent is a factor that should be considered when assessing the quantity of energy dissipated from each aromatic center. The influence of different substituents on electronic delocalization is also analyzed, as well as the role played by their frontier molecular orbitals.

## 1. Introduction

**Figure 1:**
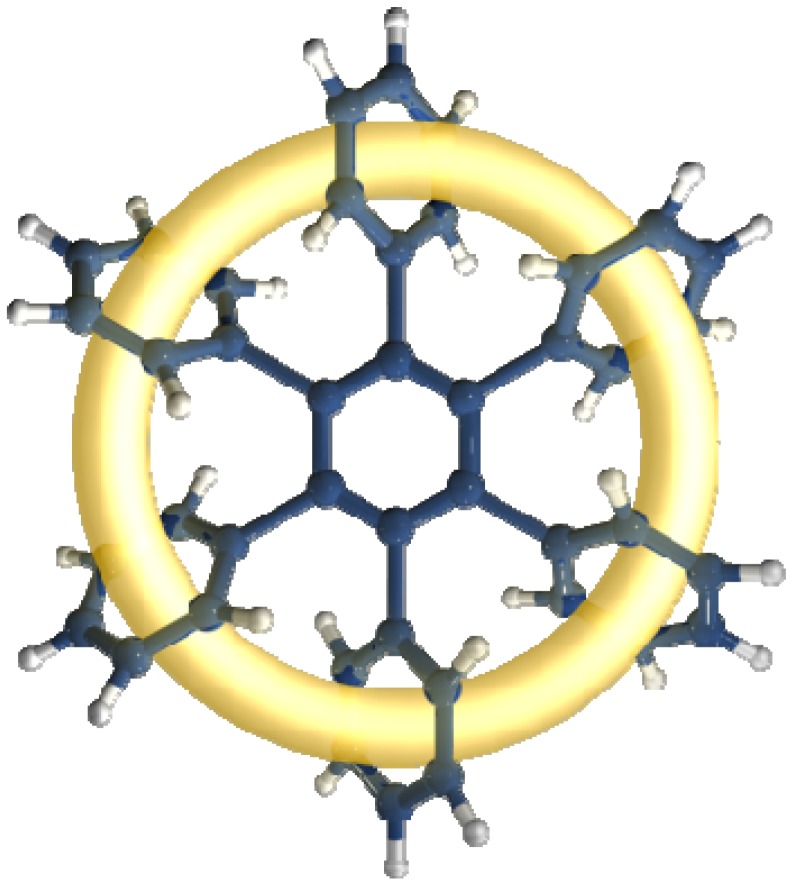
Toroidal delocalization.

The toroidal delocalization of electrons among hexaarylbenzenes (**HABs**) has been defined by a number of research groups [1,2]. **HABs** have been regarded as one of the most important structures for demonstrating this phenomenon and are expected to manifest very particular electronic features [3] because they are able to adopt a propeller-like conformation which enables π-π interactions [4,5]. These can induce an effect which gives place to the electronic dispersion on a large ring, comprising all involved substituents (see Figure 1).

This new phenomenon has been assessed in order to design molecular devices [6] for studying electric currents in Mobius systems [7], or intramolecular charge-transfer systems [8,9].

All **HAB** derivatives may serve as model compounds for studying this peculiar phenomenon, as any one of these systems has six arene units that are able to assume a ring-like arrangement, where each lateral ring is positioned at an angle of approximately 60° with respect to the central ring [2]. Thus each lateral ring has a sloping position with respect to the others, and in this way each ring has a view of the sloping center of the two continuous rings, also enabling communication by which the electrons can move.

Considering that in all cases, the central ring as well as the lateral rings consist of aromatic fragments, toroidal delocalization should thus be caused by the dissipation of these electronic currents [7], and thus aromaticity of the entire system should undergo strong modifications when compared to free arenes. These modifications can be electron release or electron withdraw depending on the direction that the inductive effect gives to each particular case and should vary according to the intrinsic aromaticity of each species and this is related to several factors, for example electronic resonance or stabilization energy. In general, the aromaticity studies have been carried out by means three different criteria, *i.e.*, the magnetic criterion represented by all the NICS methods developed by Schleyer and co-workers [10], the geometric criterion presented by Kruszewski and Krygowski [11] and several energy criteria. The isodesmic reactions method developed by our group belongs to the last classification and was chosen because the energy differences are expected to be very strong for the molecules under study. 

This work aims to quantify the energy dispersion caused by the lack of aromaticity that results from toroidal delocalization; therefore several **HAB** systems have been studied, registering several structural and electronic differences. The central aromatic ring, as well as the six peripheral substituents contributes to pi-electron delocalization with a net electronic charge. Thus the intention here is to carry out a quantification of this dispersed energy, whereby the energy change is estimated by defining the difference between aromaticity of the **HAB** species and the corresponding value in their constituent fragments. 

The energy values for all the species, *i.e*., the **HAB** molecules studied here, as well as the neutral constituent fragments, were evaluated with reference to the Aromatic Stabilization Energy (ASE) [12] as yielded by isodesmic reactions specifically designed for these purposes. Therefore, the differences previously mentioned were gotten in each case by subtracting the obtained value for the isolated constituents from each **HAB** molecule. Another useful factor for evaluating the lack of electrons from the aromatic rings refers to the full Mulliken charges values; therefore a similar study was carried out, subtracting the charge values of the **HAB** molecules from their constituent fragments, with the intention of revealing an approximate electron quantity transferred from the rings to the toroidal current. It is possible to discover other electronic factors with potential for collaborating with electronic dispersion (for example conjugation with the central ring), however, this other possible source of dispersion would be the same for all cases being studied because all of these compounds belong to the same model, *i.e.*, an hexasubstituted central ring. Thus an approximation of the energy values derived from electronic toroidal delocalization will be considered. The nature of the frontier molecular orbitals in all cases is also analyzed because it is important to consider a possible pathway for the electronic flux, with particular reference to the HOMO- LUMO set and the related orbitals.

## 2. Methods

All calculations were carried out by applying a pure DFT method for energy evaluations with Becke’s gradient corrections [13] for exchange, and Perdew-Wang’s for correlation [14]. This is the scheme for the B3PW91 method which forms part of the GAUSSIAN09 [15] package. In all cases, the base was 6-31G**. This basis set was chosen in order to compare our results with published values [16]. All geometries were optimized and corresponding frequency calculations were carried out in all cases in order to prove the absolute findings for energy minima; all the optimized geometries in form of Cartesian coordinates are available in the supporting information. Specific isodesmic reactions [17,18] were designed for each case, some of the cases mainly those of a number of parent molecules can be considered to comprise homodesmotic reactions in terms of the new classification [19], however in the majority of **HAB** derivative cases, reactions are only isodesmic.

## 3. Results and Discussion

The **HAB** molecules that were studied in this work have the same structure in all cases, consisting of a central benzene ring completely substituted by different groups. The set of studied compounds was divided into three large groups. In the first one, derivatives with large toroidal dissipation are considered. In the second one, derivatives with short toroidal dissipation are included. In the last one, derivatives containing pyrene are considered as a special case. 

The intention of this study is to search for an indicator of the electron mobility because toroidal delocalization requires that the electronic clouds intrinsic to each lateral aromatic ring be shared; therefore the proposed study will apply an energy criterion, *i.e.* one that evaluates ASE, or rather the differences between the different ASE values. 

Thus a thermochemical study is necessary, where isodesmic reactions will be designed for each particular case and the intrinsic ASE of each species evaluated. Subsequently, the corresponding result is subtracted six times from the isodesmic reaction, which relates to the free substituent. 

The idea is to carry out an energy analysis, taking the entire energy pertaining to the large molecules and comparing it to the energy pertaining to the free substituents. Thus the difference should indicate the dissipated energy manifested in toroidal delocalization. Notably, the value obtained for the free substituents is added to −21.2 kcal/mol; this result corresponds to the estimation of aromaticity referring to the central aromatic ring, as reported in the original work by George [17,18]. This strategy will be the same for all the studied cases. 

### 3.1. Parent Molecules

The study consist in the design of the isodesmic reactions searching for the parent molecules, where the presence of double bonds for different carbon atoms resembles the combinations required for particular products, as well as the presence of heteroatoms in a particular presentation. Therefore the designed reactions contain almost identical parent molecules. All the parent molecules are described in the following schemes.

(a) 3,4-Divinyl-1,3,5-hexatriene (C_10_H_12_). 


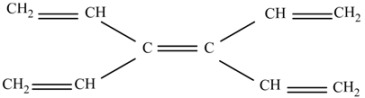


This molecule is an excellent CC double bond source, and is also useful because it contains the C atoms without hydrogen substituents, which are important for describing the central rings.

(b) 3-Nitropropene (C_3_H_5_NO_2_). 


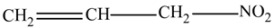


This molecule contains the CC double bonds and the NO_2 _group, simultaneously, this molecule is restricted in the fundamental feature of electronic resonance, however, it is an excellent source of the NO_2_ group with an organic bond fragment which is essential for the nitro aromatic substituents.

(c) Vinylacetonitrile (C_4_H_5_N).


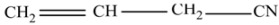


This is similar to the last example, but the molecule contains also the CN bond.

(d) 2-Ethenyl-1,3-butadiene (C_6_H_8_).


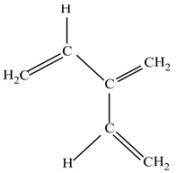


This is a molecule with several terminal CC double bonds and the central C atom without hydrogen.

(e) Di(2-nitrovinyl)-di(2-carboxylvinyl) hydrazone (C_12_H_10_N_4_O_8_). 


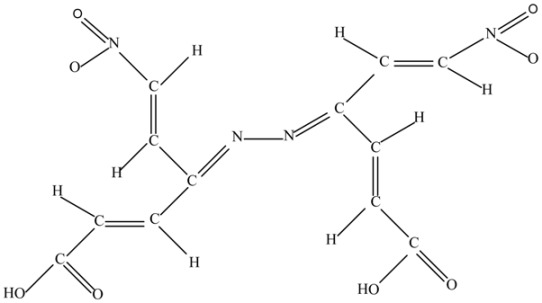


This molecule manifests the combination characteristic of the parent molecule a) tetravinylethylene (TVE), and b) it is a source of the nitrogen atoms.

(f) Acrylic acid (C_3_H_4_O_2_).


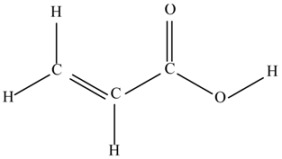


This molecule is a source of the CC and CO double bonds and CO joints of two types of groups: C=O and COOH. 

(g) 1,3-Butadiene (C_4_H_6_). 


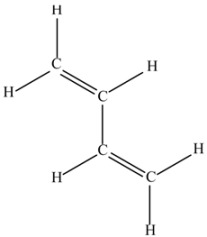


This molecule shows simple conjugated olefinic groups.

(h) *cis*-3,4-Di(2-nitrovinyl)-1,3,5-hexatriene-1,6-dicarboxylic acid (C_12_H_10_N_2_O_8_).


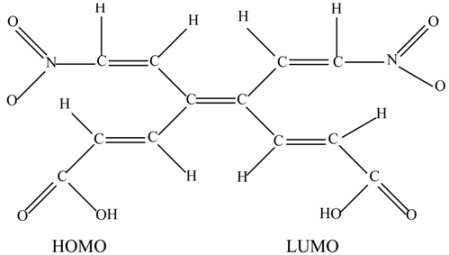


The molecule is rich in the C=C, NO_2_ and COOH fragments.

Notably, there are several cases among the set of the parent molecules presented above, where it is possible that different isomers may be included. However, in the isodesmic reactions, only the isomers shown here were taken into consideration, in order to obtain congruent results. Isomers were chosen, not for their greater stability but rather because they manifested optimal geometry for participating in the designed reactions.

Likewise, an analysis of the electronic mobility taking advantage of the Mulliken charge results, yielded by the same method of calculation, was carried out. The analysis was made by simply subtracting the charges in the free substituents from those in the substituted items; the results of these differences should represent the lack of electrons from the primitive rings that go to the toroidal delocalization in the resultant complex molecules. This analysis is presented after the energy results for each case.

### 3.2. Analysis of Results

#### 3.2.1. Molecules with Marked Toroidal Delocalization

**Figure 2:**
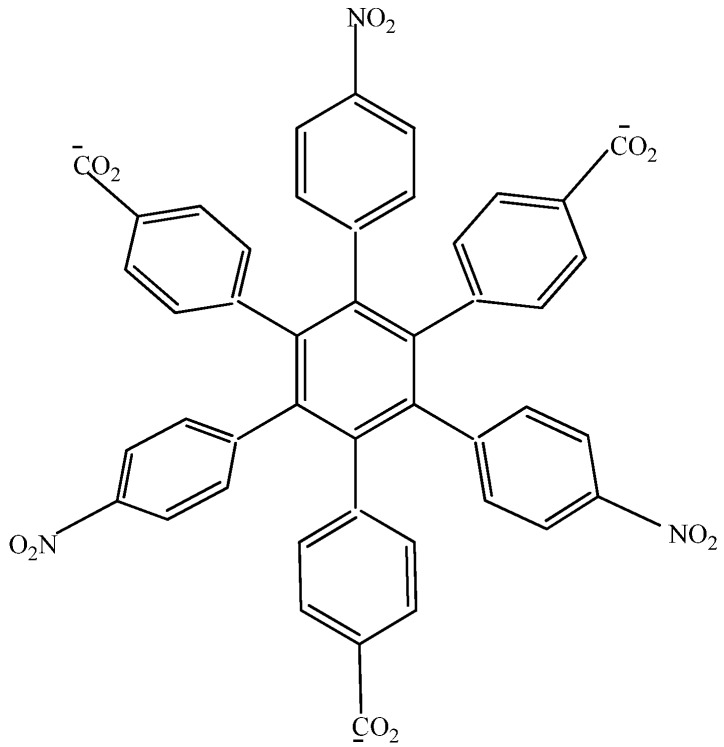
Structure of **HAB1**.

The first studied case was **HAB1** (Figure 2) containing three 4-nitrophenyl and three (4-carboxylate) phenyl (benzoate) fragments.

The corresponding isodesmic reaction for **HAB1** is:

*42C_4_H_6_ + 3CH_10_ → 2C_45_N_3_O_12_H_27 _+ 57C_2_H_4_* (1)

The corresponding isodesmic reaction for benzoate anion is:

*7C_4_H_6_ + 2C_3_H_4_O_2_ → 2C_7_H_5_O_2_ + 10C_2_H_4_* (2)

Whereas the same for nitrobenzene is:

*4C_3_H_5_NO_2_ + 3C_10_H_12_ → 4C_6_H_5_NO_2_ + 9C_2_H_4_* (3)

**Figure 3:**
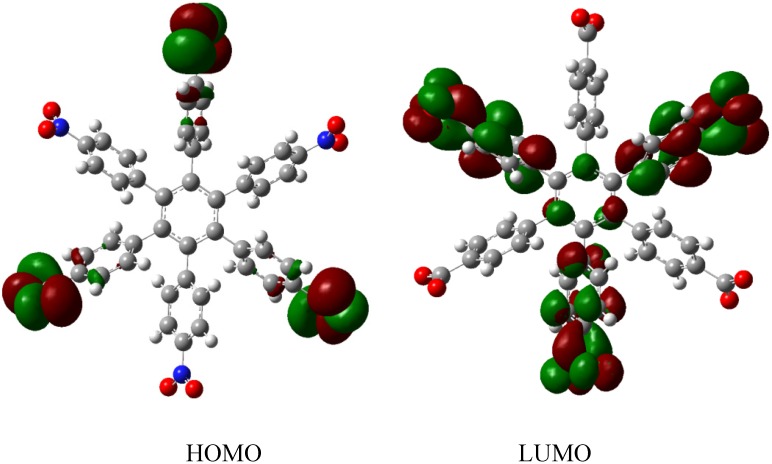
Frontier molecular orbitals of **HAB1**.

Therefore, the value of the energy for **HAB1** is −139.62 kcal/mol; we made a subtraction of this result and the set of three (4-carboxylate) phenyl anions and three nitrobenzene molecules (plus 21.1 kcal/mol from the central ring) and the result is 69.72 kcal/mol. Notably this result was achieved, assuming three negative charges on the original molecule, as this factor would generate electron mobility. Similarly, it is evident that the energy gap between HOMO and LUMO is too short compared with organic semiconductor species [20], with a value of 1.007 eV, although this result is derived from the fact that the molecular orbitals drastically change their position because of the presence of the negative charges. The shape of the molecular orbitals is presented in Figure 3.

The nature of the molecule is reflected in these molecular orbitals, where the HOMO produces the entire negative charge coming from the electron pairs and correspondingly the LUMO operates as an acceptor region centered on the 4-nitrophenyl substituents. It appears that this represents a very good target for toroidal delocalization, because the frontier molecular orbitals are localized in different zones of the molecule and the energy gap is too short, therefore, it is expected that the electrons flow easily between them, even though this behavior obeys the ionic nature of the species, a feature that is altered in the next example.

The power to generate low energy toroidal dissipation was investigated in order to discover whether it might be caused by strong inductive effect or be totally independent of this. Thus a molecule was designed, hypothetically manifesting very strong electron withdrawal activity, in order to study the inductive effect where the electron-withdrawal characteristic is very dominant. 

**Figure 4:**
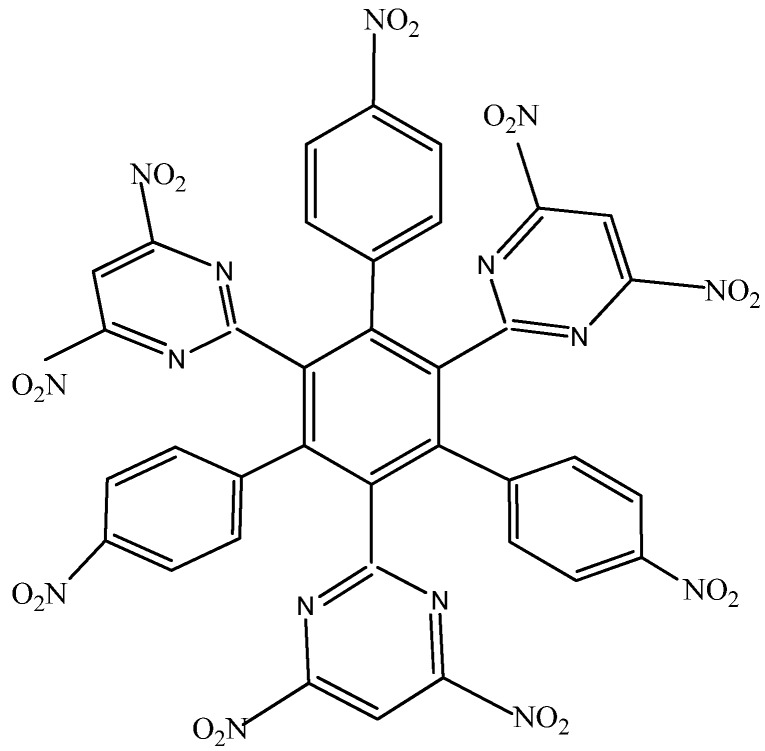
Structure of **HAB2**.

**HAB2** consists of three 4-nitrophenyl and three 4,6-dinitro-2-pyrimidyl fragments and its shape is presented in Figure 4. The azines are a species known for manifesting very strong electron-withdrawal effect [21], so this kind of fragment is a good target for illustrating the influence that these phenomena have on electronic mobility.

The corresponding isodesmic reaction for **HAB2** is:

*33C_4_H_6_ + 15C_12_H_10_N_4_O_8_ → 4C_36_H_15_N_15_O_18_ + 48C_2_H_4_ + 24C_3_H_4_O_2_* (4)

Whereas the isodesmic reaction for 4, 6-dinitropyrimidine is:

*3C_2_H_4_ + C_12_H_10_N_4_O_8_ → C_4_H_2_N_4_O_4_ + 2C_4_H_6_ + 2C_3_H_4_O_2_* (5)

**Figure 5:**
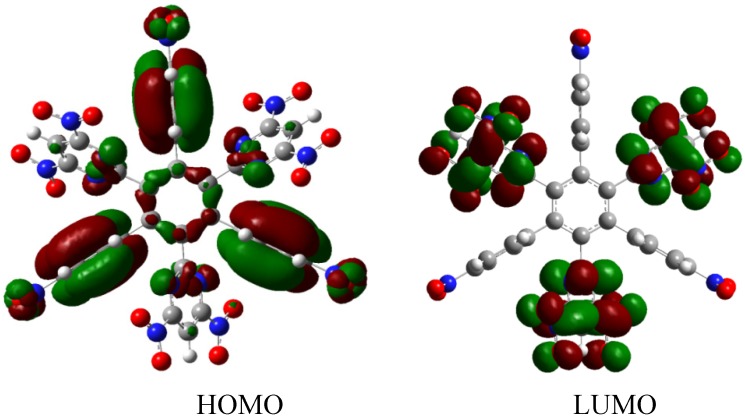
Frontier molecular orbitals for **HAB2**.

The behavior of this molecule is very similar to that found in the previous example; the homodesmotic reaction for the **HAB2** is −92.118 kcal/mol, whereas the result for three diazine (−40.16 kcal/mol) plus three nitrobenzene molecules and the central ring is 118.16 kcal/mol with the same alternative frontier molecular orbitals (Figure 5), the nature of these molecular orbitals depends on the presence of the diazine substituent because the strong electron withdrawal effect caused that the LUMO was totally localized on this fragment yielding an electron flow to this zone of the molecule. However, in this instance there are no negative charges, in contrast this molecule is totally attractive to electrons, a fact reflected in the energy gap between HOMO and LUMO, which is 3.61 eV, suggesting weak semiconductor behavior. However the similarities in behavior between both molecules are evident in the marked energy for toroidal delocalization, suggesting that the alternation of certain specific substituents may account for the improvement in electronic flow. 

**Figure 6:**
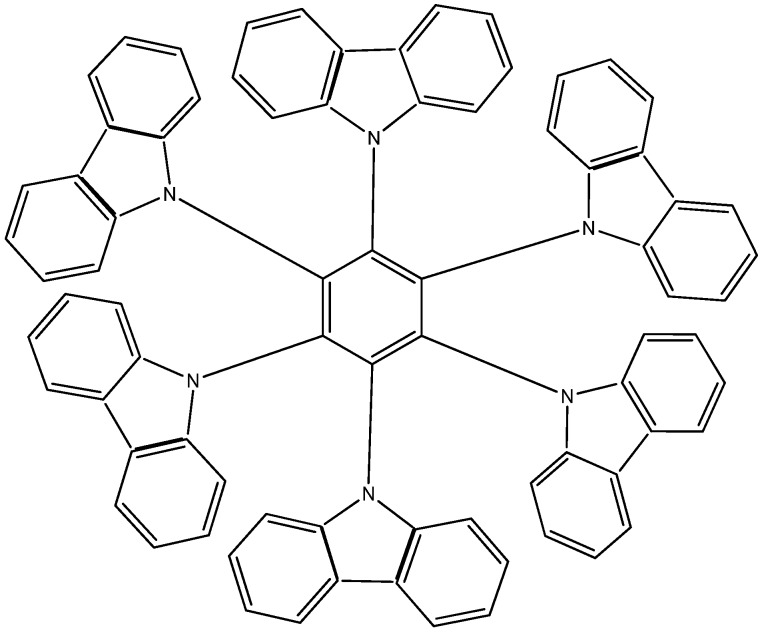
Structure of **HAB**.

The third example **HAB3** corresponds to the molecule proposed by Lambert [2,22], where six 9-carbazolyl substituents surround the central ring (see Figure 6).

*24C_4_H_5_N + 45C_10_H_12_ → 4C_78_H_48_N_6_ + 117C_2_H_4_* (6)

This is the isodesmic reaction for **HAB3**, whereas the corresponding reaction for dibenzenepyrrole is:

*2C_4_H_5_N + 3C_10_H_12_ → 2C_12_H_9_N + 7C_2_H_4_* (7)

The results of the energy are −432.29 kcal/mol for **HAB3**, and −79.69 kcal/mol for dibenzenpyrrol. Therefore, applying the same mechanism, the result of the energy obtained for toroidal dissipation in this instance is 66.95 kcal/mol.

**Figure 7:**
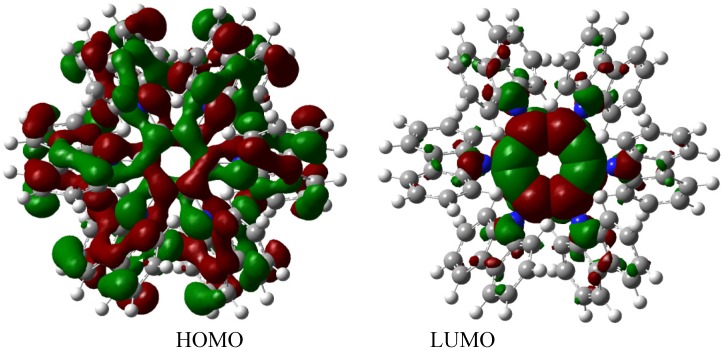
Frontier molecular orbitals for **HAB3** derivative.

The description of this molecular orbital shares certain characteristics with previous examples because there are three molecular orbitals contributing to the set of the HOMO in an accidental degeneration process, this strong concentration, that is shown in Figure 7, causes a massive electronic flux on this wave function. However, the LUMO has a very low value (−0.06eV) and the energy gap between frontier molecular orbitals is 3.87 eV; once again demonstrating weak semiconductor behavior. However, in this case, the nature of the LUMO is very different because the greatest concentration of the wave function is found near the central ring and perhaps for this reason the value for toroidal dissipation is so different than those manifested by the last molecule considering that the electrons have the tendency to remain in the central ring and not in the toroidal dispersion. 

The behavior of the last three molecules is more or less similar, because all are notable for the substantial energy that they display, indicating toroidal delocalization and semiconductor behavior (or conductor behavior in the case of the ion). However, the situation is not clear in terms of inductive effect, as it must be important to consider the idea of alternation of fragments manifesting varied electronic behavior, if optimum situations for revealing interesting electronic features are to be designed.

#### 3.2.2. Molecules Manifesting Limited Toroidal Delocalization

**Figure 8:**
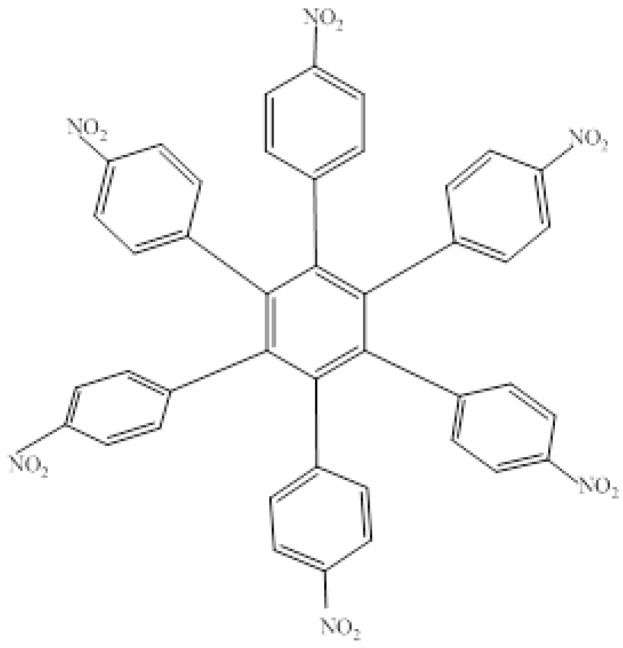
Structure of **HAB4**.

The proposal concerning the influence of inductive effect on the generation of extensive or limited toroidal delocalization effects was explored, presenting two molecules with totally contrasting behavior; the first one consisting of a benzene ring totally substituted by the 4-nitrophenyl groups, and the second one, by the 4-aminophenyl groups. The idea is to carry out a similar study to that conducted for the last cases, and then compare the results to a large electron withdrawal species (hexa(4-nitrophenyl)benzene) and an example of total electron release (hexa(4-aminophenyl)benzene). However, there appears to be no clear relationship between the inductive effect and toroidal dissipation, as contrarily both molecules manifest almost the same behavior with the respect to the electronic dissipation, it seems that the presence of a substituent with a known inductive effect can affect the direction of the electronic flux considering electron release or withdraw, but do not affect the quality of this flux The results are as follows: 

The first molecule to be studied from this set was **HAB4**, containing six 4-nitrophenyl groups (see Figure 8), and the corresponding isodesmic reaction is presented in Equation (8). 

*24C_3_H_5_NO_2_ + 27C_10_H_12_ → 4C_42_H_24_N_6_O_12_ + 87C_2_H_4_* (8)

##### Isodesmic Reaction for **HAB4**

The corresponding isodesmic reaction for the nitrobenzene molecule is presented above (Equation (3)). It is a known fact that the 4-nitrophenyl group is one of the most aromatic electron withdrawing species [23], therefore this case is expected to represent an outstanding example of electronic transportation. The result for toroidal delocalization makes a very interesting contrast, when compared with electron-donor examples. The energy value for **HAB4** is −128.95 kcal/mol, and this result subtracted from six nitrobenzene molecules and the central ring (−158.5 kcal/mol) yields 29.57 kcal/mol for the toroidal dissipation. This result is very low when compared with the molecules analyzed above, suggesting that the best targets for designing electronic active molecules must be electron release substituents, a possibility that will be analyzed later. However, the results relating to this molecule deserve careful analysis in order to establish the real nature of these derivatives and their potential application.

**Figure 9:**
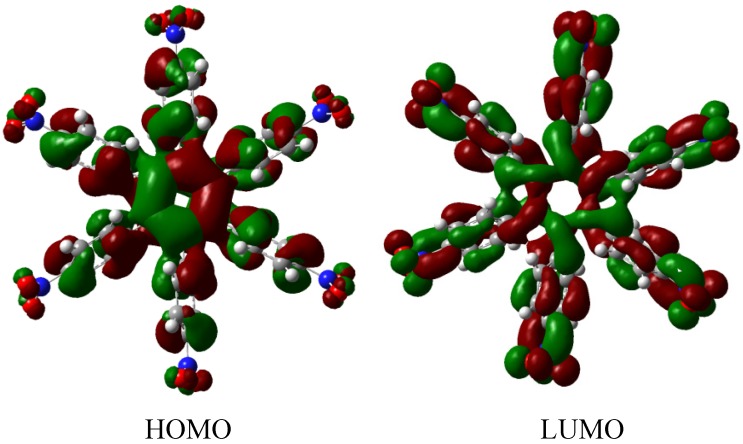
Frontier molecular orbitals for **HAB4**.

The molecular orbital scheme indicates a very interesting situation because both orbitals are in the same molecular zone, causing that the electronic flow exists between the frontier molecular orbitals; the shape of which are depicted in Figure 9.

However, the behavior will not be as good as this scheme predicts because the energy gap between HOMO and LUMO is 4.32 eV, suggesting insulator behavior and as both frontier molecular orbitals occupy the same probabilistic regions; this predicts serious difficulties in terms of electronic transportation, leading to the conclusion that this does not represent a good example of an active electronic molecule.

**Figure 10:**
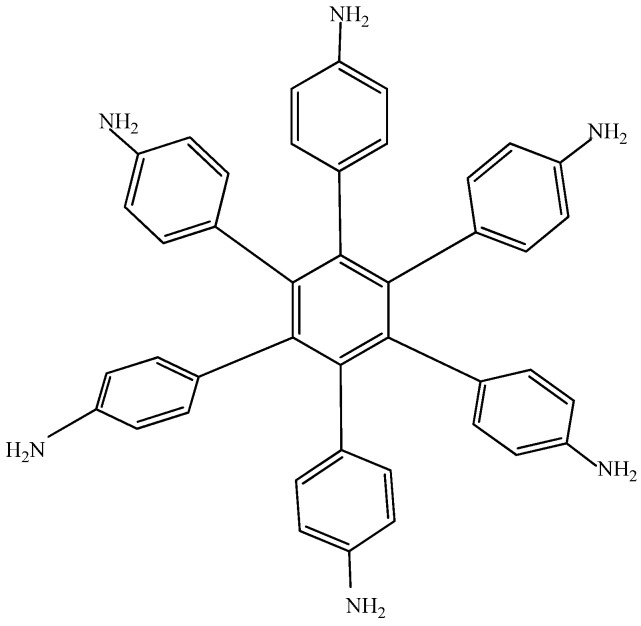
Structure of **HAB5**.

Electronic-withdrawal substituents represent predictably bad targets in contrast to molecules surrounded by electron release substituents, which comprise very good targets. For this reason, **HAB5** was designed. This species contains six 4-aminophenyl substituents and shows very interesting results. The shape of **HAB5** is presented in Figure 10.

The isodesmic reaction for **HAB5** is as follows:

*24C_4_H_5_N + 15C_10_H_12_ → 4C_42_H_36_N_6_ + 39C_2_H_4_* (9)

Whereas the isodesmic reaction for evaluating aniline is presented in equation 10. 

*4C_4_H_5_N + C_10_H_12_ → 4C_6_H_7_N + C_2_H_4_* (10)

The result of the energy is −264.49 kcal/mol for **HAB5**, and −44.23 kcal/mol for the aniline molecule, therefore considering the central ring and six 4-nitrobenzene molecules, the toroidal dissipation for this molecule is 21.99 kcal/mol, indicating a very similar situation to that found in the previous example. However, the nature of the substituents is completely different because in this case the amino substituent causes the electron release effect. Likewise, the different origin and the values for the inductive effect are not sufficient to make a significant difference, thus it seems to be imperative to have regions with very similar energy *i.e.* short energy gap between HOMO and LUMO, where the flux of the electrons is able to take place and where electrons are very mobile.

**Figure 11:**
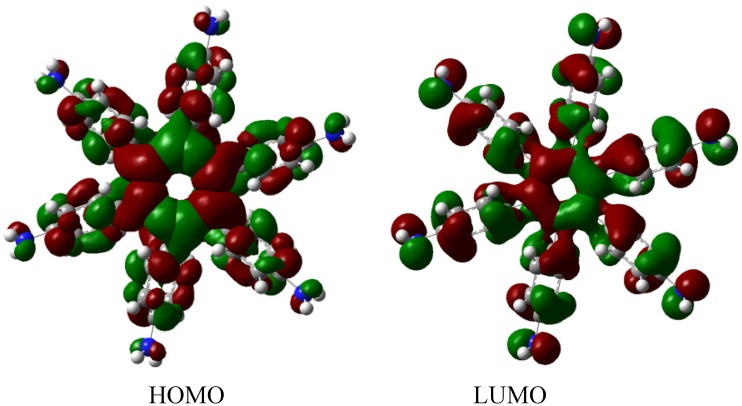
Frontier molecular orbitals for **HAB5**.

The molecular orbital distribution indicates an unusual situation because the molecule in the frozen structure belongs to the C_6_ point group, a group that has no triple degenerated irreducible representations; however the distribution in the HOMO, HOMO-1 and HOMO-2 manifests such minor differences that there is a suggestion of triple accidental degeneration. LUMO is a positive molecular orbital and the energy gap between HOMO and LUMO is 4.51 eV, once again indicating insulator behavior, therefore, the energy gap seems to be fundamental in order to explain the electronic dissipation because large gaps lead to the lack of relationship between the frontier molecular orbitals. Therefore, an important point is the nature of the shape of these molecular orbitals, which are presented in Figure 11.

These distributions suggest that the electronic flux should be guaranteed because there are important concentrations in both frontier molecular orbitals, however, the large energy gap gives place to a lack of relationship between the mentioned orbitals as in **HAB4**; thus it would appear that an electronic flow between these two functions is improbable. 

**Figure 12:**
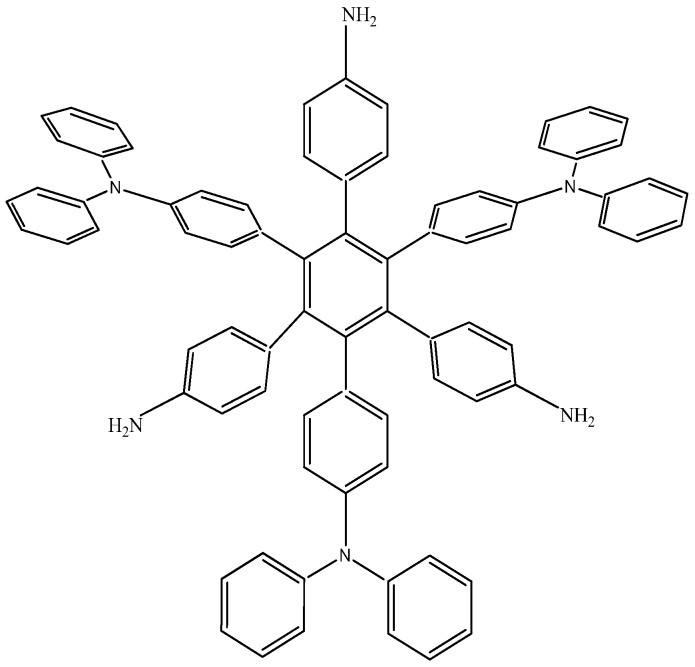
Structure of **HAB6**.

The last example in this section shows a combination of electron-donor substituents in an attempt to confirm the last hypothesis; it has three 4-aminophenyl groups and three 4-(diphenylamino)phenyl groups and was denominated **HAB6** (see Figure 12).

The isodesmic reaction for **HAB6** follows:

*24C_4_H_5_N + 39C_10_H_12_ → 4C_78_H_60_N_6_ + 87C_2_H_4_* (11)

Whereas the isodesmic reaction for triphenylamine is:

*4C_4_H_5_N + 9C_10_H_12_ → 4C_18_H_15_N + 17C_2_H_4_* (12)

**Figure 13:**
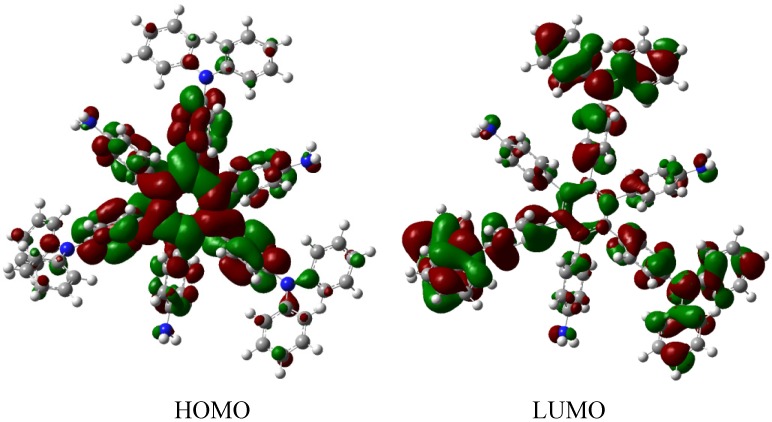
Frontier molecular orbitals for **HAB6**.

The energy result for this reaction is −97.21 kcal/mol. Therefore, considering this and the other results, the last values are −423.54 kcal/mol for the **HAB6**, −443.53 kcal/mol for the three aniline (see equation 10), and three triphenylamine plus −21.1 from the central ring with a resulting toroidal dissipation value of 19.76 kcal/mol. Considering these results of this molecule it seems that the combination is not appropriated for the electronic dispersion and this feature can be explained with reference to the molecular orbital analysis and the shape of HOMO and LUMO that is shown in Figure 13.

The situation of the molecular orbitals for **HAB6** is somewhat different to that indicated in the other examples. Similar behavior to that of hexa(4-aminophenyl) benzene (**HAB5**) is predicted because it only contains electron donor substituents. However, the HOMO shows that (4-diphenylamino) phenyl substituents are likely to be present, mainly with the aromatic ring joined to the central ring whereas the 4-aminophenyl orbitals indicate medium participation. In contrast, the LUMO indicates participation on the part of all substituents, including the central ring. Therefore the description suggests that electronic flow goes from an electron donor (*i.e.*, the (4-diphenylamino) phenyl fragments) towards the periphery and also towards the central regions. This phenomenon is the result of combining two types of electron donor species; however, it seems that the mixing of two donors does not result in a very good generator of toroidal delocalization, as the energy gap in this case is 4.08 eV, once again suggesting insulator behavior. Thus it appears that electronic flow is inhibited when fragments are of the same nature. 

#### 3.2.3. Molecules Containing the Pyrenyl Groups

**Figure 14:**
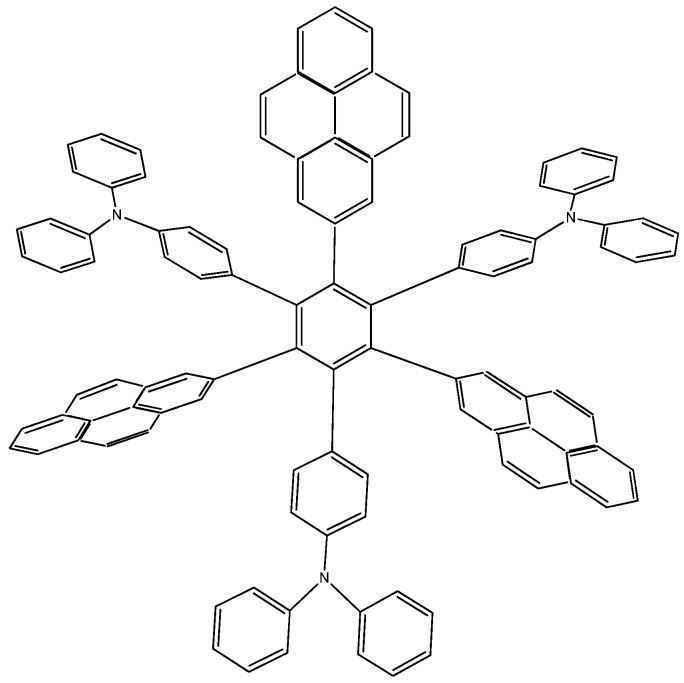
Structure of **HAB7**.

Pyrene is one of the most peculiar polyaromatic hydrocarbons [24,25]. It would appear to have different aromatic regions within its perimeter and to be able to work either as an electron donor or as an electron acceptor depending on the environment. It has particular electronic characteristics and is also known to be a very strong carcinogenic agent. This chameleonic fragment is likely to be useful for designing OLED’s [26,27] and is thus likely to be apt for designing good toroidal delocalization derivatives; indeed this idea is proposed by Lambert [9]. Due to the ambiguous nature of this molecule, it can act either as an electron acceptor or donor, so that the molecule proposed by Lambert, as well as two more locally designed species were studied here. Results contrast with those presented above; the first example **HAB7**, that contains three 2-pyrenyl and three (4-diphenylamino)phenyl substituents (Figure 14). This molecule is precisely that provided by Lambert.

The corresponding isodesmic reaction for **HAB7** is:

*12C_4_H_5_N + 69C_10_H_12_ → 4C_108_H_69_N_3_ + 153C_2_H_4_* (13)

Whereas the isodesmic reaction for pyrene is:

*4C_6_H_9_ + 9C_10_H_12_ → 4C_16_H_10_ + 25C_2_H_4_* (14)

**Figure 15:**
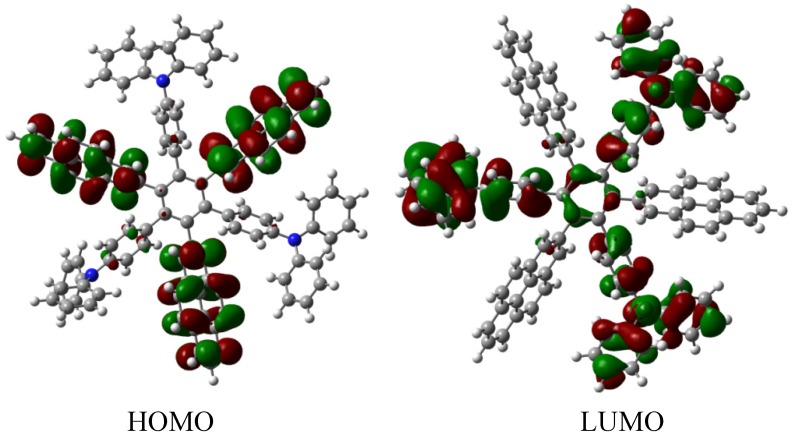
Frontier molecular orbitals for **HAB7**.

The energy result for **HAB7** derivative is −599.58 kcal/mol, is subtracted from −612.213 kcal/mol, representing three pyrene and three triphenylamine molecules plus the central ring, and yielding 12.63 kcal/mol for the toroidal delocalization result. This value is not as good as that obtained for **HAB5**; however, it deserves to be analyzed as an example of frontier molecular orbitals seeking for a good orbital combination that promotes electronic transfer. The frontier molecular orbitals are presented in Figure 15.

The frontier molecular orbitals manifest very different distributions; the only possibility in the case of the HOMO relates to the (4-diphenylamino) phenyl substituents with no other participation, whereas the LUMO is formed entirely from the wave functions derived from the 2-pyrenyl fragments. This behavior is very similar to the case of the electron withdrawal substituents studied above. The curious factor lies in the fact that the energy gap for this molecule is 3.2, eV, representing the best value obtained in these calculations for a set of neutral molecules, although it has the smallest value for toroidal dissipation, concurring with Lambert’s results, thus indicating that the molecule has good characteristics in terms of an active electronic species [9]. This result establishes hope for the other cases because a small value for toroidal dissipation will not have great impact, if the molecular orbitals help to generate good communication for electronic transfer.

**Figure 16:**
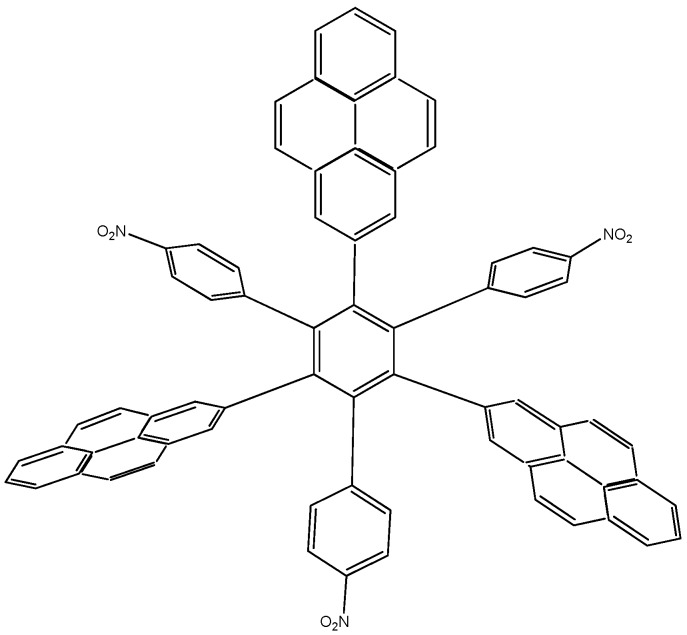
Structure of **HAB8**.

Another example was designed with the 2-pyrenyl fragment in the molecules, with the intention of including an electron-withdrawal fragment. This feature was used to evaluate possible influence in terms of electron mobility in this type of group, and thus pyrene was combined with nitrobenzene (**HAB8** derivative), once again giving an intriguing result. The shape of the molecule is depicted in Figure 16.

The corresponding isodesmic reaction for **HAB8** is:

*12C_3_H_5_NO_2_ + 51C_10_H_12_ → 4C_72_H_39_N_3_O_6_ + 129C_2_H_4_* (15)

The energy results are similar to the previous example, −365.77 kcal/mol for this molecule that is subtracted from −390.06 kcal/mol, derived from the substituents and the central ring, so that the result for toroidal dissipation is 24.29 kcal/mol; a value that is very similar to that for **HAB4**.

**Figure 17:**
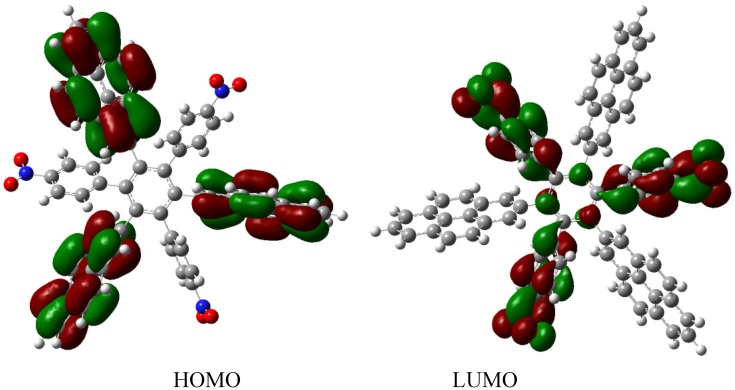
Frontier molecular orbitals for **HAB8**.

The molecular orbitals scheme is notorious in the case of **HAB8** (see Figure 17), because of the particular chemistry of pyrene [24]. In LUMO, the only probabilities are derived from the 4-nitrophenyl fragments together with the central ring, whereas the HOMO only has the probability of the 2-pyrenyl fragments themselves, this behavior is the opposite to that mentioned above in Figure 15 for the case of **HAB7** with the (4-diphenylamino) phenyl and 2-pyrenyl groups. This is predictable due to the contrary nature of these ligands, one manifesting electron-release activity and the other electron-withdrawal activity. This description can also be useful because electronic flow is directed entirely from the HOMO which contains the 2-pyrenyl fragments towards the LUMO with the 4-nitrophenyl substituents; the important point being that the energy gap between HOMO and LUMO for this case is 2.84 eV, a value which reveals strong semiconductor character and guarantees the electronic flow between all the lateral substituents. Thus this instance is very similar to that of other molecules containing pyrene, *i.e.*, a low energy gap, alternation between frontier molecular orbitals and moderate electron toroidal delocalization.

**Figure 18:**
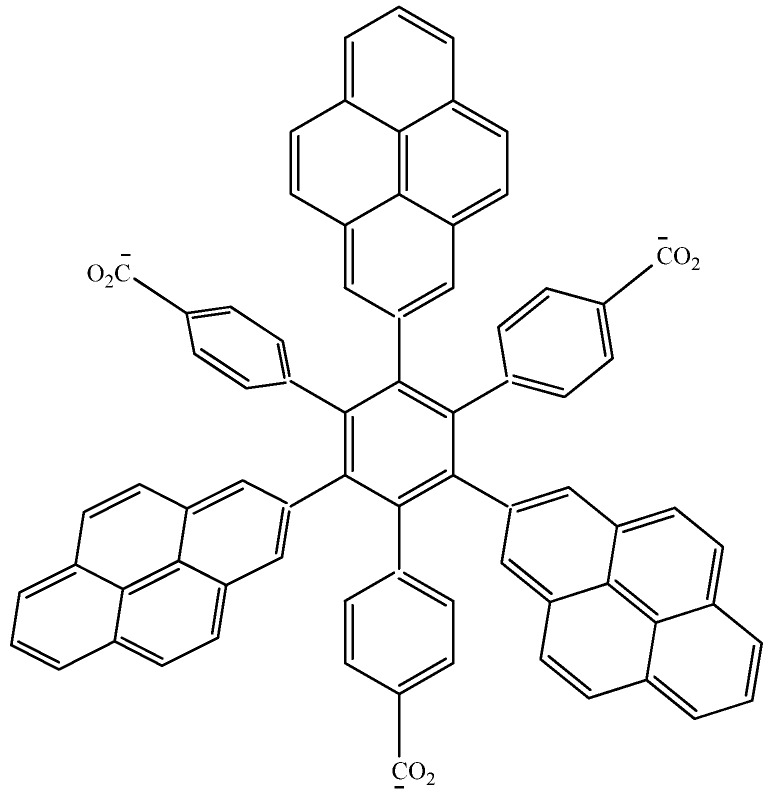
Structure of **HAB9**.

In order to assess these last assertions, a new molecule (**HAB9**), containing pyrene was designed, in this case, the 2-pyrenyl fragment was accompanied by the (4-carboxylate) phenyl one, and the shape of the resulting molecule is presented in Figure 18.

The corresponding isodesmic reaction for **HAB9** is:

*105C_10_H_12_ + 24C_3_H_4_O_2_ → 8C_75_H_39_O_6_ + 261C_2_H_4_* (16)

The isodesmic reaction yields the energy value of −389.21 kcal/mol, whereas the value of three pyrene molecules, plus three (4-carboxylate) phenyl anions, plus the central ring is −440.902 kcal/mol, which again is subtracted from the value cited above; therefore the toroidal electronic delocalization energy is 51.692 kcal/mol, a greater value than that for the last two examples.

**Figure 19:**
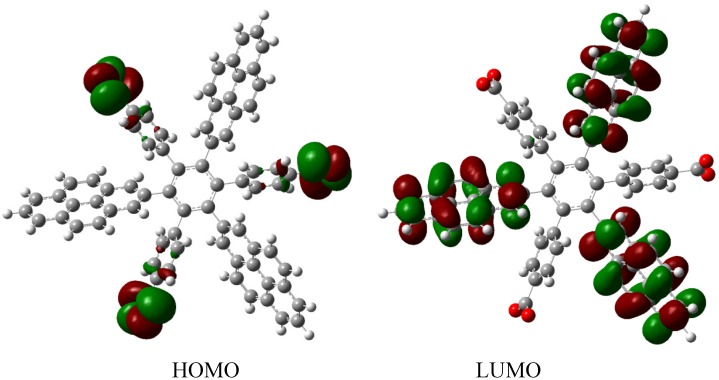
Frontier molecular orbitals for **HAB9**.

The frontier molecular orbitals for **HAB9** (see Figure 19), manifest the same behavior as the other pyrene derivatives, a HOMO that supports the negative charge on the carboxylate surface and a LUMO that is completely localized on the 2-pyrenyl fragments, however the energy gap for this case in 1.74 eV, a predictably narrow value, owing to the three negative charges.

The behavior of the molecule is very similar to that of its counterparts; conductor species, isolated frontier molecular orbitals and narrow energy gap. However, it is important to note the role played by the 2-pyrenyl fragment, in the present case this represents a very pure probability function localized on the LUMO, but in the last description where it is complemented by the 4-nitrophenyl group, it works as a HOMO, besides the fact that the carboxylate group bears a negative charge causes that the HOMO is practically completely localized on this radical. However, in the other example where the pair is (4-diphenylamino) phenyl substituent, the 2-pyrenyl fragment is once again localized in the LUMO. This indicates the chameleonic behavior cited above, pyrene adapts easily to the environment and works as a donor when faced with an acceptor, or as an acceptor when faced with a donor. In conclusion, it seems to be a good idea to include this fragment or something similar when dealing with this kind of molecule, because it guarantees some kind of electron mobility. An interesting report exists describing how a **HAB** derivative containing the 2-pyrenyl fragment was prepared [28], where the compound shows very interesting spectroscopic properties and indeed manifests a strong and red-shifted fluorescence, a fact suggesting interesting behavior. However, in the present study this was not included because in this particular species there are no other fragments that can serve as electronic complements.

It is also important to note that a very interesting report on hexa(4-aminophenyl)benzene has been written describing its interesting electronic and spectroscopic characteristics [1]. This fact is important in terms of the type of study presented here, because the electronic characteristics of this molecule were shown to constitute one of the most discrete in the entire context, therefore it is probable that the behavior manifesting stronger electronic manifestations can be addressed in experimental manner in the near future. 

A curious detail worthy of comment is that the HOMO-LUMO energy gap for all cases was achieved and compared in Table 1, where notably all the electron donor substituent species are shown to be insulators, furthermore the compound totally substituted by 4-nitrophenyl fragments indicates the same behavior, even though the NO_2_ derivative is a strong electron acceptor. Moreover, all the species containing the 2-pyrenyl fragment manifest the behavior of semiconductors, whereas the one containing NO_2_ and diazine appears to be a borderline species. All charged molecules manifest marked conductor behavior. This feature should be taken into account because the capacity for electron transfer (low energy gap) is also important for designing a good material, in spite of the magnitude of the energy corresponding to toroidal dissipation. 

**Table 1:** Comparison among HOMO-LUMO energy gap of the **HAB** derivatives under study.

Substituents	HOMO-LUMO Gap (in eV)
4-Aminophenyl (**HAB5**)	4.38
4-Nitrophenyl (**HAB1**)	4.307
4-Aminophenyl, (4-diphenylamino) phenyl (**HAB6**)	4.084
9-Carbazolyl (**HAB3**)	3.841
4-Nitrophenyl, (4,6-dinitro-2-pyrimidyl) (**HAB2**)	3.62
2-Pyrenyl, (4-diphenylamino) phenyl (**HAB7**)	3.205
4-Nitrophenyl, 2-pyrenyl (**HAB8**)	2.836
2-Pyrenyl, (4-carboxylate-phenyl)(**HAB9**)	1.747
4-Nitrophenyl, (4-carboxylate-phenyl) (**HAB4**)	1.007

The global Mulliken charges values (from selected fragments) can be useful for assessing the donation of electrons from the substituents (and even from the central ring) to the toroidal delocalization. These values were estimated in a way similar to those for ASE, *i.e.* the sum of local charges of the aromatic rings on the **HAB’s** substituted species were subtracted from the same values derived from the free molecules, the results are known as relative charge and are presented in Table 2.

**Table 2:** Comparison among relative charges of the **HAB** derivatives under study.

Substituents	Relative Charge
4-Aminophenyl (HAB5)	2.71
4-Nitrophenyl, (4,6-dinitro-2-pyrimidyl) (HAB2)	2.26
9-Carbazolyl (HAB3)	1.96
4-Nitrophenyl, (4-carboxylate-phenyl) (HAB4)	1.64
4-Aminophenyl, (4-diphenylamino) phenyl (HAB6)	1.37
2-Pyrenyl, (4-diphenylamino) phenyl (HAB7)	0.98
4-Nitrophenyl (HAB1)	0.96
2-Pyrenyl, (4-carboxylate-phenyl) (HAB9)	0.89
4-Nitrophenyl, 2-pyrenyl (HAB8)	0.68

From a summary of the phenomena, it is possible to suggest the best targets for compounds with potential electronic device application: in first place those compounds bearing a negative charge, obviously show a narrow energy gap, good energy dissipation and reasonable charge values; these can therefore be considered for this classification. The other cases present different situations; the species containing diazine or carbazole seem to be the best neutral targets, whereas those containing pyrene are also good in terms of gaps and charges and finally the more symmetrical cases with NO_2_ and NH_2_ are not as good as the other cases, possibly because of their marked symmetry and also taking into account that they fall into the category of extreme inductive effect. Thus this collection of phenomena is likely to indicate a more symmetric distribution of electrons, resulting in lack of mobility.

Indeed it would appear that a good target must constitute a molecule that fulfills several requirements; firstly manifesting toroidal dissipation, even though the energy magnitude of this should not constitute an important factor. However, this characteristic is very important for enabling electronic transit. Secondly, it should have a narrow HOMO-LUMO gap, although this may be larger than that of the classical representation of conductor, semiconductor and insulator species. Thirdly, it should manifest effective alternation in terms of the frontier molecular orbitals, as well as availability of electrons and/or holes to facilitate flow.

## 4. Conclusions

The thermochemical value of the electron toroidal delocalization for several **HAB** derivatives was obtained by carrying out calculations for isodesmic reactions. This value can vary, depending on the different aromaticities of the fragments but there are several other factors to consider when proposing effective targets for the design of electronically active molecules, first the nature and separation of the frontier molecular orbitals are very important because the phenomenon of toroidal dissipation is present mainly in species with strong relation between HOMO and LUMO, *i.e.*, the presence of free electrons (in HOMO) or holes (in LUMO), the better electronic interchange with short energy gap indicates optimum targets. Second, the distribution of relative charges also leads as an appropriate indicator of the electronic dissipation. The electronic properties of the derivatives studied in this work open the opportunities for their application in the design of electronic devices as OLED’s.
